# Microscopic Invasions, Prognoses, and Recurrence Patterns of Stage I Adenocarcinomas Manifesting as Part-Solid Ground-Glass Nodules: Comparison With Adenocarcinomas Appearing as Solid Nodules After Matching Their Solid Parts’ Size

**DOI:** 10.1097/MD.0000000000003419

**Published:** 2016-04-18

**Authors:** Eui Jin Hwang, Chang Min Park, Young Tae Kim, Hyungjin Kim, Jin Mo Goo

**Affiliations:** From the Department of Radiology (EJH, CMP, HK, JMG), Seoul National University College of Medicine; Institute of Radiation Medicine (EJH, CMP, HK, JMG), Seoul National University Medical Research Center; Deparment of Radiology (EJH), Armed Forces Seoul Hospital; Cancer Research Institute (CMP, HK, JMG), Seoul National University; and Department of Thoracic Surgery and Cardiovascular Surgery (YTK), Seoul National University College of Medicine, Seoul, Korea.

## Abstract

The purpose of the present study was to compare the frequency of microscopic invasions, disease-free-survival (DFS), and the frequency and pattern of disease recurrence between stage I pulmonary adenocarcinomas appearing as solid nodules and those appearing as part-solid ground-glass nodules (GGNs) after matching their solid parts’ size (*D*_solid_) and patients’ age.

Among 501 patients who underwent curative surgery for stage I pulmonary adenocarcinomas between 2003 and 2011, 172 patients (86 with solid nodules [M: F = 36: 50; mean age, 62.8 years] and 86 with part-solid GGNs [M:F = 30:56; mean age, 63.0 years]) matched for *D*_solid_ and patients’ age were included. DFS, frequency of microscopic invasions, recurrence, and recurrence pattern were compared between the two groups.

No significant difference was observed in the frequency of microscopic invasions between the two groups (visceral pleural invasion, 30.23% vs. 29.07%, *P* = 0.867; lymphatic invasion, 5.81% vs. 3.49%, *P* = 0.720; vascular invasion, 1.16% vs. 0%, *P* = 1.000; solid nodules vs. part-slid GGNs, respectively) and DFS (estimated 5-year DFS, 83.6% vs. 81.9%, *P* = 0.744; solid nodules vs. part-slid GGNs, respectively). As for recurrence and recurrence pattern, there were no significant differences between the solid nodule group (14/86), and part-solid GGN group (12/86) (*P* = 0.670). Lung parenchymal nodules were the most frequent pattern of disease recurrence in both groups, followed by pleural seeding.

In conclusion, after matching *D*_solid_ and patients’ age, there was no significant difference in the frequency of microscopic invasions, DFS, and the frequency and pattern of recurrence between stage I pulmonary adenocarcinomas appearing as solid nodules and part-solid GGNs.

## INTRODUCTION

Lung cancer is the leading cause of cancer death worldwide,^1^ and the incidence of pulmonary adenocarcinoma, its most common histological subtype, has been shown to be increasing.^2^ With recent advances in computed tomography (CT) technology and accumulation of research on the radiological features of pulmonary adenocarcinoma, it is known that pulmonary adenocarcinomas manifest as either solid nodules or subsolid nodules [either pure ground-glass nodules (GGNs) or part-solid GGNs] on CT.^3,4^

Previous studies have reported that patients with adenocarcinomas appearing as subsolid nodules showed better prognoses than those with adenocarcinomas appearing as solid nodules, and that pulmonary adenocarcinomas containing larger ground-glass opacity (GGO) portions had better prognoses than those with smaller GGO portions.^5–9^ These observations may be attributed to the fact that the GGO portions within adenocarcinomas represent preinvasive components of pulmonary adenocarcinomas such as atypical adenomatous hyperplasia (AAH) or adenocarcinoma-in-situ (AIS) while the solid parts on CT typically indicate invasive adenocarcinoma components, pathologically.^10–12^

However, recent researches^13,14^ have pointed us toward a different approach of assessing these nodules, as they reported that solid component size (*D*_solid_) rather than whole tumor size including GGO parts (*D*_whole_) were more critical for the assessment of patients’ prognosis and thus the tumor stage should be evaluated based on the tumor's solid parts instead of the size of the entire tumor containing the GGOs. Yet, the prognosis, recurrence rate, and recurrence pattern of adenocarcinomas manifesting as subsolid nodules have not been fully evaluated nor compared with those appearing as solid nodules based on *D*_solid_.

Therefore, the purpose of our study was to investigate the frequency of microscopic invasions, disease-free-survival (DFS), and the frequency and pattern of recurrence after resection of pulmonary adenocarcinomas appearing as part-solid GGNs and to compare them with those appearing as solid nodules after matching their *D*_solid_ and patients’ age.

## METHODS

This retrospective study was approved by the Institutional Review Board of Seoul National University Hospital with a waiver of the requirement for patients’ informed consent.

### Patient Selection

Based on our electronic medical records, 642 patients were initially included in the present study, using the following inclusion criteria: patients who underwent curative surgery at our institution for stage I pulmonary adenocarcinoma between 2003 and 2011, and patients with available preoperative thin-section CT images with slice thicknesses of 1.25 mm or thinner. Among them, 141 patients were excluded due to the following reasons: patients had other synchronous or metachronous primary lung cancers (n = 88); patients had pulmonary adenocarcinomas manifesting as pure GGNs on preoperative CT (n = 35); and patients had a clinical course after surgery which was not able to be traced (n = 18). Finally, a total of 501 patients (206 male and 295 female patients; mean age, 61.8 years) with 501 pulmonary adenocarcinomas of which 304 tumors appeared as solid nodules (solid nodule group) and 197 tumors manifested as part-solid GGNs (part-solid GGN group), were included in the present study prior to matching.

### Matching

The unmatched patient population was identical to that included in our previous study.^13^ In the previous study, we found measuring *D*_solid_ were better than *D*_whole_ for prognosis prediction of adenocarcinomas appearing as part-solid GGNs and *D*_solid_ and patients’ age were independent prognostic factors in adenocarcinoma population including both solid nodule group and part-solid GGN group. In this context, in the present study, we matched the solid nodule group and part-solid GGN group using *D*_solid_ and patients’ age in a one-by-one manner to compare the prognosis, frequency of microscopic invasions and recurrences, and the pattern of recurrences between adenocarcinomas appearing as solid nodules (solid nodule group) and those appearing as part-solid GGNs (part-solid GGN group) while taking care to avoid potential confounding bias. We first divided the entire patient population into multiple subgroups according to *D*_solid_ and patients’ age, with each subgroup defined by intervals of 0.5 cm and 5 years for *D*_solid_ and age, respectively, that is, *D*_solid_ 0.1 to 0.5 cm and age 36 to 40 years, *D*_solid_ 0.6 to 1.0 cm and age 36 to 40 years, *D*_solid_ 4.6 to 5.0 cm and age over 80 years. Thereafter, equal numbers of patients for the solid nodule group and the part-solid GGN group were selected from each subgroup. For example, when solid nodules outnumbered part-solid GGNs in a certain subgroup, all part-solid GGNs were included in the final cohort, while solid nodules were randomly selected to have the same number of patients as those with part-solid GGNs using a table of random numbers. When there were more part-solid GGNs than solid nodules, patient selection was performed in the opposite manner. Finally, a total of 172 patients were included in the present study: 86 patients with adenocarcinomas appearing as solid nodules (36 males and 50 females; mean age, 62.8 years; range, 38–82 years) and 86 patients with adenocarcinomas appearing as part-solid GGNs (30 male and 56 female; mean age 63.0 years, ranges, 36–86 years).

### Clinical Data Collection

The following clinical data were collected from medical records: age and sex of the patients; date and type of surgery; dates of preoperative CT and positron emission tomography-computed tomography (PET-CT) scans; and presence and date of lung cancer recurrence. Dates of lung cancer recurrences were defined as those of the initial detection of pathologically or medically diagnosed recurrent lung cancer. Medical determination of lung cancer recurrence was made by a multidisciplinary discussion, including medical oncologists, pulmonologists, thoracic surgeons, radiation oncologists, radiologists, and nuclear medicine physicians at our institution.

Disease-free survival (DFS) was calculated from the date of surgery until either recurrence of the tumor (event) or until the date that patients underwent the latest follow-up when recurrences did not occur (censored). The mean ± standard deviation of the follow-up duration was 1500 ± 665 days (range, 189–3261 days).

### CT Acquisition and Assessment

Preoperative CT data were acquired using one of the following CT scanners: Somatom Definition, Sensation-16 (Siemens Healthcare, Erlangen, Germany), Brilliance-64 (Philips Healthcare, Eindhoven, The Netherlands), and Lightspeed Ultra (GE healthcare, Milwaukee, WI) with 120 kVP, 60 to 120 mAs. Images were reconstructed using the medium sharp reconstruction algorithm, with slice thicknesses of 1 or 1.25 mm. All CT scans were obtained with the patients placed in the supine position with full inspiration. Median time interval between preoperative CT scans and surgery was 13 days (range, 0–25 days).

For measurement, one-dimensional measurements for primary lung cancer lesions were performed by a single radiologist (EJH, with 4 years of experience in thoracic radiology). On 1 or 1.25 mm thin-section axial CT images, both the longest diameters of the whole nodule including GGO component (*D*_whole_) and those of only the solid component (*D*_solid_) were measured on independent slices. All measurements were performed using a window width and level of 1500 and −700 HU, respectively.

Radiological assessment of recurrent lung cancers was determined via consensus discussion by two radiologists (EJH, and CMP, with 4 and 14 years of experience in thoracic radiology, respectively). As for the evaluation of the pattern of cancer recurrence, the pattern of disease recurrence was categorized into the following 5 categories: pleural seeding, bronchial stump recurrence, lung nodule or mass, thoracic LN metastasis, and metastasis to distant organs.

### PET-CT Acquisition and Assessment

Preoperative ^18^F-fluorodeoxyglucose (^18^F-FDG) PET-CT scans were performed in 137 of 172 patients (79.7%). PET-CT scans were performed on dedicated PET-CT scanners (Gemini, Philips Healthcare; Biograph 40, Siemens Healthcare), after at least 6 hours of fasting, and after intravenous injection of ^18^F-FDG at a dose of 5.2 MBq/kg of body weight, 1 hour prior to the examination.

For assessment of the ^18^F-FDG uptake of primary lung cancer lesions, maximum standardized uptake values (SUV_max_) were utilized. Measurements were performed using a commercially available analysis package (Syngo.via, Siemens Healthcare). After visual identification of the area of the primary lung cancer lesion, spherical volumes of interests (VOIs) were placed to cover the entire lesion. After normalization of SUV for injection dose and body weight, SUV_max_ was obtained from the highest pixel value in the VOIs. The median time interval between preoperative PET-CT and surgery was 9 days (range, 1–76 days).

### Surgery and Pathologic Assessment

In both groups, 83 of 86 patients underwent standard lobectomy, while the other 3 underwent wedge resection.

All pathologic information was collected retrospectively from medical records, including the presence of visceral pleural invasion, lymphatic invasion, and vascular invasion. All pathologic evaluations were performed after formalin fixation of the specimen and staining with hematoxylin-eosin.

### Statistical Analyses

All statistical analyses were performed with IBM SPSS Statistics (version 21.0, IBM, Armonk, NY). *χ*^2^ tests and Fisher exact tests were performed for categorical variables and the Student *t* test for continuous variables. Kaplan–Meier analyses with logrank tests were performed for the evaluation and comparison of survival. Results with *P* values <0.05 were considered to indicate a statistically significant difference.

## RESULTS

Table 1 shows the demographic, clinical, and radiologic features of the 2 groups. There were no significant differences in age, sex, type of surgery, follow-up duration, and *D*_solid_ between the solid nodule group and the part-solid GGN group.

**TABLE 1:**
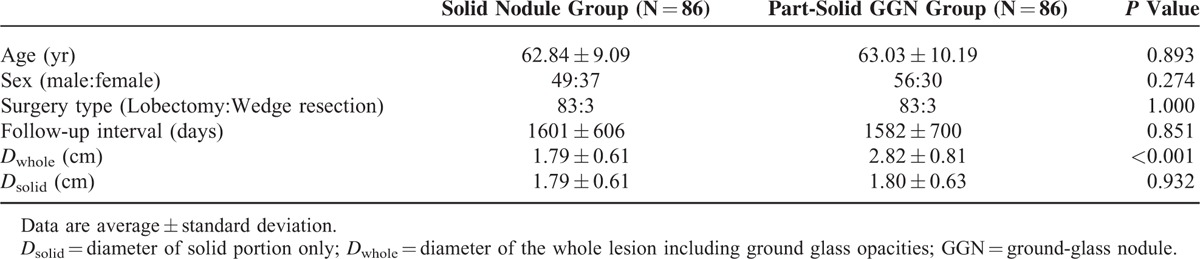
Comparison of Demographic, Clinical and Radiological Features Between the Solid Nodule Group and Part-Solid GGN Group

### Comparison of the Frequency of Microscopic Invasions

Table 2 shows the frequencies of visceral pleural invasion, lymphatic invasion, and vascular invasion on histopathologic diagnosis. There were no significant differences in the frequencies of microscopic invasions between the solid nodule group and part-solid GGN group.

**TABLE 2:**
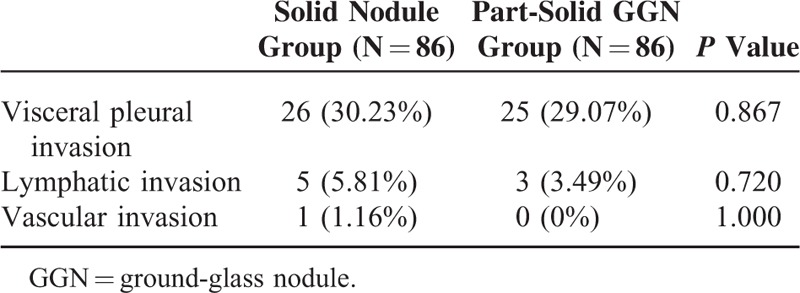
Frequency of Microscopic Invasions

### Comparison of Disease-Free Survival and Patterns of Recurrence

Among the 172 patients, 26 patients (15.1%) comprised of 14 patients in the solid nodule group and 12 in the part-solid GGN group were determined to have postoperative disease recurrence. Six patients were pathologically confirmed to have disease recurrence and the remaining 20 patients were determined through multidisciplinary discussion.

**FIGURE 1:**
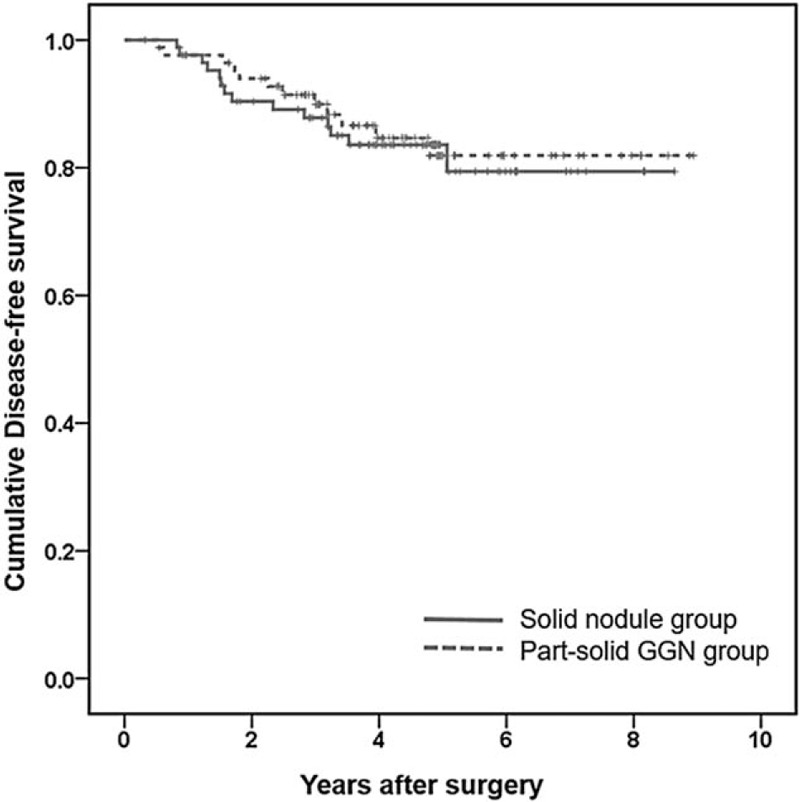
Kaplan–Meier survival curves for DFS of the solid nodule group (red) and part-solid GGN group (green). No statistically significant difference was observed between the 2 groups.

There were no statistically significant differences in DFS between the 2 groups (Logrank test, *P* = 0.744, Figure 1). Averaged DFS for the solid nodule group and the part-solid GGN group were 2703 days (95% confidence interval, 2484–2923 days) and 2803 days (95% confidence interval, 2641–3061 days), respectively. Estimated 5-year DFS were 83.6% and 81.9%, respectively.

There were also no statistically significant differences in the frequency of disease recurrence between the 2 groups (16.3% in the solid nodule group and 14.0% in the part-solid GGN group, *P* = 0.670). Mean ± standard deviation of time intervals between surgery and disease recurrence were 799 ± 477 days for the solid nodule group and 890 ± 472 days for the part-solid GGN group (*P* = 0.620). Table 3 shows the numbers of each recurrence pattern. There were no statistically significant differences in recurrence patterns between the 2 groups. In both groups, lung nodules were the most common pattern of recurrence (50%; 7 of 14 in the solid nodule group and 6 of 12 in the part-solid GGN group), followed by pleural seeding (6 of 14 in the solid nodule group; 5 of 12 in the part-solid GGN group). Among the 6 patients in the part-solid GGN group, whose recurrence patterns were pulmonary nodules, 2 patients showed multiple GGNs, while the other 4 patients showed multiple solid nodules.

**TABLE 3:**
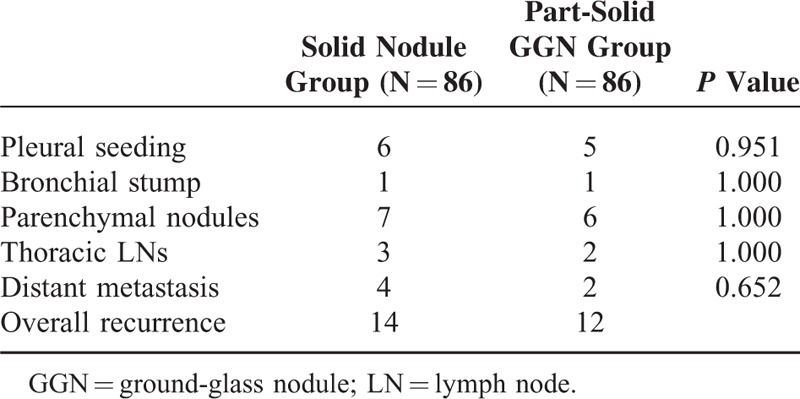
Comparison of the Patterns of Recurrence Between the Solid Nodule Group and Part-Solid GGN Group

**FIGURE 2:**
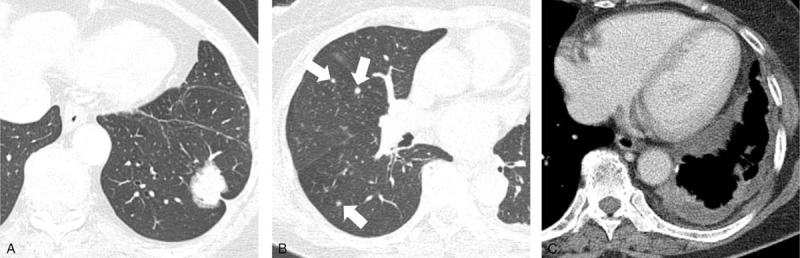
A 67-year-old female patient who underwent left lower lobectomy for pulmonary adenocarcinoma manifesting as a part-solid GGN. A, Preoperative chest CT shows a part-solid GGN in the left lower lobe of the lung. The whole nodule including GGO parts and its solid part were measured as 3.1 and 2.4 cm, respectively. B, C, Follow-up chest CT performed 4 years and 10 months after surgery shows multiple solid nodules in both lungs (arrows) and irregular pleural thickening with effusion.

**FIGURE 3:**
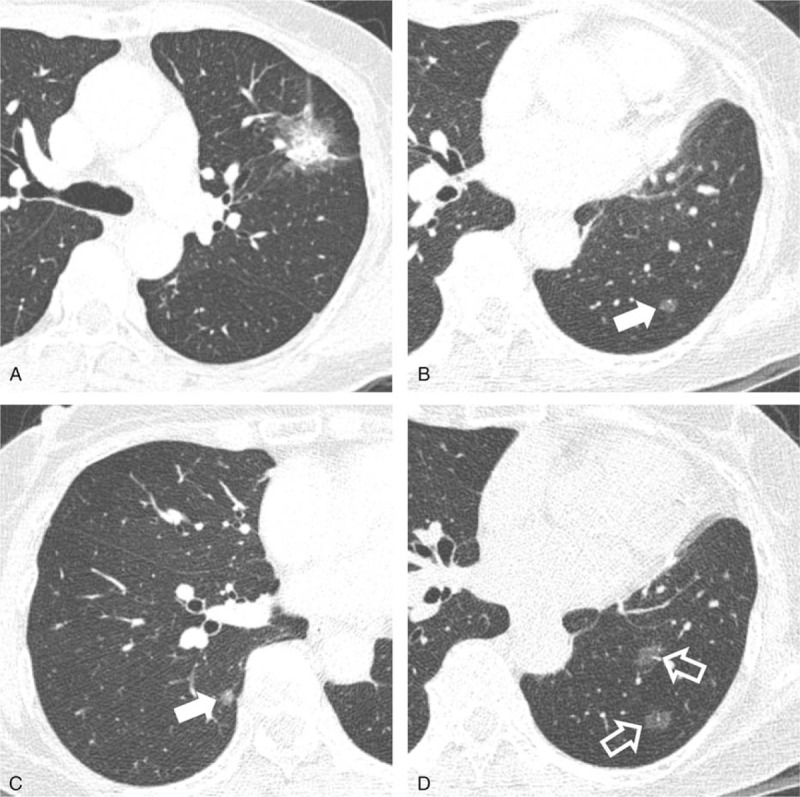
A 69-year-old female patient who underwent left upper lobectomy for a part-solid GGN-type adenocarcinoma. A, Preoperative chest CT shows a part-solid GGN in the right upper lobe of the lung. The whole nodule including GGO parts and its solid part were measured as 3.0 and 1.8 cm, respectively. Visceral pleural invasion and lymphatic invasion were observed on pathologic examination. B, C, Follow-up chest CT performed 2 years after surgery shows multiple pure GGNs in the bilateral lungs (arrows). D, Subsequent follow-up chest CT performed 6 months later than (B) shows an increased size and number of multiple pure GGNs (open arrows), which were determined as metastases through multidisciplinary discussion.

Table 4 shows the clinical and radiological characteristics of patients who showed recurrence in the part-solid GGN group. Figures 2 and 3 show their representative cases.

**TABLE 4:**
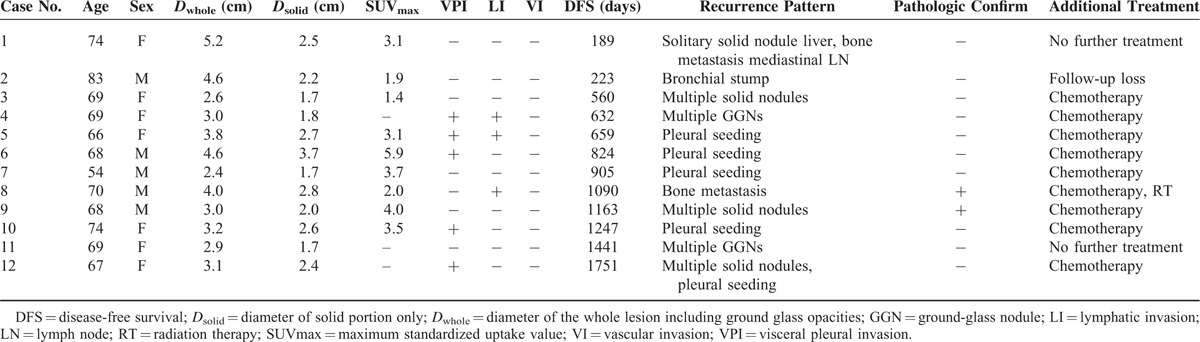
Clinical and Radiological Characteristics of Patients Who Showed Recurrence in the Part-Solid GGN Group

### Comparison of ^18^F-FDG PET-CT Findings

A total of 68 patients of 86 (79.1%) underwent preoperative PET-CT scans in the solid nodule group, compared with 69 patients (80.2%) in the part-solid GGN. Significantly higher SUV_max_ were observed in the solid nodule group (4.22 ± 3.04) than those in the part-solid GGN group (3.03 ± 2.45) (*P* = 0.013). However, there were no significant differences in *D*_solid_ (mean ± standard deviation *D*_solid_, 1.86 ± 0.64 cm vs. 1.85 ± 0.66 cm, *P* = 0.931) and DFS (mean DFS, 2500 days vs. 2885 days, *P* = 0.511) between the solid nodule group and the part-solid GGN group among those who underwent preoperative PET-CT scans.

## DISCUSSION

In the present study, we compared the tumor behavior of pulmonary adenocarcinomas appearing as solid nodules and those appearing as part-solid GGNs after matching their *D*_solid_ and patients’ age, and found no statistically significant difference in terms of the frequency of microscopic invasions, DFS, and frequency and patterns of disease recurrence between the 2 groups.

Matching is a widely used method to control confounding variables in a cohort study.^15,16^ It has been previously shown that *D*_solid_ and patients’ age were independent significant prognostic factors for DFS,^13^ and our aim in the present study was to investigate whether adenocarcinomas manifesting as part-solid GGNs with similar solid parts’ size (*D*_solid_) would exhibit similar or different clinical behavior from pulmonary adenocarcinomas appearing as solid nodules. Meanwhile, because of retrospective design of our study, matching additional variables may result in smaller sample size and decreased statistical efficiency. For this purpose, we matched the *D*_solid_ and patients’ age between both groups, and found that among 501 initially included patients (304 patients in the solid nodule group and 197 in the part-solid GGN group), 172 patients (34.3%, 87 patients in both groups) were able to be included for analyses after the matching process.

Microscopic invasion of pulmonary adenocarcinomas, including visceral pleural invasion, lymphatic invasion, and vascular invasion, has been reported to be an independent risk factor for disease recurrence and a marker for the malignant behavior of tumors.^17–20^ In the present study, no difference in the frequency of microscopic invasions was observed between the solid nodule and part-solid GGN group. Thus, it can be stated that adenocarcinomas manifesting as part-solid GGNs show no significantly different tumor behavior from those appearing as solid nodules, when their *D*_solid_ are similar to those of solid nodule type adenocarcinomas. However, in a previous study employing a similar design published by Tsutani et al,^21^ solid nodules were reported to show more frequent associations with microscopic invasions even after matching *D*_solid_. We believe that this difference might be due to the difference with regard to our respective study populations. In our study, we included patient with stage I (T1a, T1b, T2a), while Tsutani et al included stage IA (T1a, T1b) adenocarcinoma only.

As for prognosis after surgery, we were able to find no significant differences in DFS between the 2 groups. In the previous study by Tsutani et al,^21^ in contrast to the results of our present study, part-solid GGNs showed significantly better DFS than solid nodules even after matching *D*_solid_. Once again, the differences in our respective study populations may explain the different prognostic results observed in our studies. In the present study, the average *D*_solid_ was 2.3 cm, while that in the study by Tsutani et al was 1.8 cm. One recent study revealed that larger *D*_solid_ could affect a greater negative influence on prognosis in patients with part-solid GGN.^13^ When *D*_solid_ was smaller than 2 cm, part-solid GGNs showed significantly longer DFS than solid nodule-type adenocarcinomas, while no significant differences were observed when *D*_solid_ was greater than 2 cm.^13^

Until now, there have been no investigations on the postoperative recurrence pattern of pulmonary adenocarcinomas appearing as part-solid GGNs. Our study revealed that lung nodules were the most frequent manifestation of recurrent adenocarcinoma in both the part-solid GGN group as well as the solid nodule group, followed by pleural seeding and distant organ metastasis; and there were no significant differences in the postoperative recurrence pattern between the 2 groups. According to previous studies, distant metastases have been reported as the most frequent pattern of recurrences.^22^ However, intrathoracic recurrences accounted for the majority of disease recurrences in the present study, which could be explained by the fact that we only included stage I lung cancers in our study population.

Interestingly, 2 patients in the part-solid GGN group showed recurrence with multiple GGNs in both lungs in our study. Multiple pure or part-solid GGNs of both lungs have been thought to be multiple primary adenocarcinomas rather than metastases and to grow slowly with an indolent clinical course.^23–25^ However, in our 2 cases, multiple GGNs appeared after surgery and grew rather rapidly, and thus those lesions were determined as metastases rather than multiple primary adenocarcinomas. Indeed, there was a previous case report which reported multiple lung metastases of pulmonary adenocarcinoma presenting as GGNs.^26^ Although it is not quite common, multiple GGNs could be one of the unique features of recurrence in part-solid GGN adenocarcinomas.

Many studies have suggested that ^18^F-FDG PET-CT scans can be effective in the prognostic assessment of pulmonary adenocarcinomas.^27–30^ In addition, several previous studies have suggested that PET-CT can also be helpful in the prognosis prediction of part-solid GGN adenocarcinomas.^31,32^ According to a previous study by Tsutani et al,^21^ solid nodules showed significantly higher SUV_max_ than part-solid GGNs and showed worse prognosis even after matching the size of their solid components. In their study, however, no significant difference in prognosis was observed between the 2 groups after matching both the solid component size and SUV_max_. In our study, significantly lower SUV_max_ was observed in the part-solid GGN group; however, no significant difference in DFS was observed. Further studies are warranted to confirm whether the difference in SUV_max_ is truly related to a different prognosis or whether it reflects the different tumor biology between solid nodule adenocarcinomas and part-solid GGN adenocarcinomas.

Finally, in addition to the intrinsic shortcomings of a retrospective study, the present study has several limitations that need to be mentioned. First, a relatively small number of patients were included in our study. Even though over 500 patients were initially included, only 86 patients in each group were analyzed after matching. Furthermore, tumor recurrences were observed in only 26 patients (15.1%). This small number of tumor recurrences has the potential to lead to a type II statistical error and our results revealing that there were no significant differences in disease recurrence between the solid nodule group and part-solid GGN group might not necessarily mean equality between the 2 groups. Further studies with a larger study population and more events with longer follow-ups may be required to confirmatively demonstrate whether the prognosis and clinical behavior of these 2 phenotypes of pulmonary adenocarcinomas are indeed equivalent when *D*_solid_ and patient age are matched. Second, a substantial number of cancer recurrences (20 of 26) were not confirmed pathologically. Third, all patients did not undergo preoperative PET-CT examinations. However, there were no significant differences in *D*_solid_, DFS and other demographic features between the 2 groups even when only patients undergoing preoperative PET-CT were included.

In conclusion, after matching *D*_solid_ and patients’ age, no significant differences were observed in the frequency of microscopic invasions, DFS, and the frequency and pattern of recurrence after surgery between pulmonary adenocarcinomas appearing as solid nodules and part-solid GGNs.
